# Long-term effectiveness of adolescent brief tobacco intervention: a follow-up study

**DOI:** 10.1186/1756-0500-5-101

**Published:** 2012-02-16

**Authors:** Antti J Saari, Jukka Kentala, Kari J Mattila

**Affiliations:** 1 University of Tampere, Tampere FI-33014, Finland; 2 Vaasa Health Care Center, Vaasa, Finland; 3 Center of General Practice, Pirkanmaa Hospital District, Tampere FI-33521, Finland

## Abstract

**Background:**

Brief tobacco intervention has been used in promoting smoking cessation and preventing the initiation of smoking. We used a cohort born in 1979 (n = 2 586) from four cities in Finland. Those born on odd days received up to four brief tobacco interventions during their annual school dental check-ups in 1992-1994 (at the age of 13-15). Those who were born on even days were used as a control group. In 2008 a follow-up questionnaire was sent to the cohort. The aim of this study was to ascertain the long-term effectiveness of brief tobacco intervention given in dental health care during school age.

**Findings:**

Responses were received from 529 people in the intervention group and 491 in the control group. In the intervention group and control group by the age of 29 there were 15.3% and 18.5% smokers respectively. This difference was not statistically significant. The difference between groups was similar to that observed when they were 14 years old.

**Conclusions:**

Brief tobacco intervention performed in dental health care in adolescence did not show effectiveness in the long-term follow-up. This type of intervention alone is insufficient to prevent smoking but supports other anti-smoking activities.

**Trial Registration:**

This study was registered at http://clinicaltrials.gov (NCT01348646).

## Background

Tobacco smoking is the most significant preventable risk factor of illness and premature death in Finland as in many other industrialized countries [1]. Adolescents in Finland start smoking early and the amount of tobacco products they use is greater than in other European countries [2,3]. Most adult smokers started to smoke in adolescence, typically between the ages of 12 and 15 years, so smoking in adolescence increases the risk of smoking in adulthood [4,5].

Adolescents are well aware of the harmful effects of smoking, but they tend to underestimate these effects and their own vulnerability [6]. The synergy of a variety of different anti-smoking strategies is necessary in order to prevent adolescent smoking [7-9]. Brief tobacco intervention has been shown to have an effect in promoting smoking cessation among adolescents [10]. Brief intervention executed in a clinical setting is the foundation of many evidence-based treatment guidelines [11-13].

During the period 1992-1994 a study was carried out in Southern and Central Ostrobothnia, in the towns of Vaasa, Pietarsaari, Kokkola and Seinäjoki [14]. These towns form a province that has very homogenous school conditions. The study tested the brief tobacco intervention method during routine dental checkups for children aged 13-15 years. This is the age at which adolescents usually start experimenting with tobacco products [5,15]. The primary aim of the study was to prevent the initiation of smoking. The cohort (n = 2,582) was divided into two groups based on their dates of birth. Those who were born on odd days (n = 1,348) received the brief intervention(s), the rest (n = 1,238) were assigned to the normal care group. Both groups responded to questions and a questionnaire about their smoking and attitudes towards smoking.

The brief intervention tested was based on the hypothesis that adolescents of this age are often very particular about small details in their appearance. Thus, the brief intervention stressed the cosmetic impact of smoking. The intervention comprised annually inquiring about smoking, showing photographs of the harmful effects of smoking on the teeth, allowing participants to examine their own mouths in a mirror, and finally counselling them in accordance with their answer to the question on smoking habits. Non-smoking adolescents received positive feedback for being non-smokers. The duration of a single brief intervention was 2-3 min [16]. The dental checkups were repeated similarly for those remaining in the study (Table 1). A participant in the intervention group received a brief intervention every year, up to four times. The number of smokers at the initial checkup was 5.7% (n = 145). The result of this research was that the brief interventions reduced the prevalence of smoking by 3 percentage points compared to the control group when both groups were 14 years old. This difference was not statistically significant. The analysis at the age of 15 was excluded due to intolerable loss of participants to the last two follow-ups [14].

**Table 1:** Numbers of participants and checkups

		Control group (n = 1 238)		Intervention group (n = 1 348)		Average age
		
	Year	n	% of group	n	% of group	**y**.
*Dental checkup*						

First session	1992	1,238	100	1,348	100	13.1

Second session	1993	1,029	83.1	1,149	85.2	14.2

Third session	1994	726	58.6	845	62.7	15.2

Fourth session	1994	247	20.0	305	22.6	15.6

*Follow-up*	2008	491	39.7	529	39.2	~29

The intervention was performed while the adolescents were forming their image of smoking and smokers. Although it was not initially found effective, we thought the intervention might affect the adolescents' smoking behavior later in life, after possible experimentation with smoking in adolescence, because the intervention promoted the development of a non-smoking self-image.

Our hypothesis was that there should be fewer adult smokers in the intervention group. The aim of this study was to ascertain the long-term effectiveness of the brief intervention described above.

## Methods

**Figure 1:**
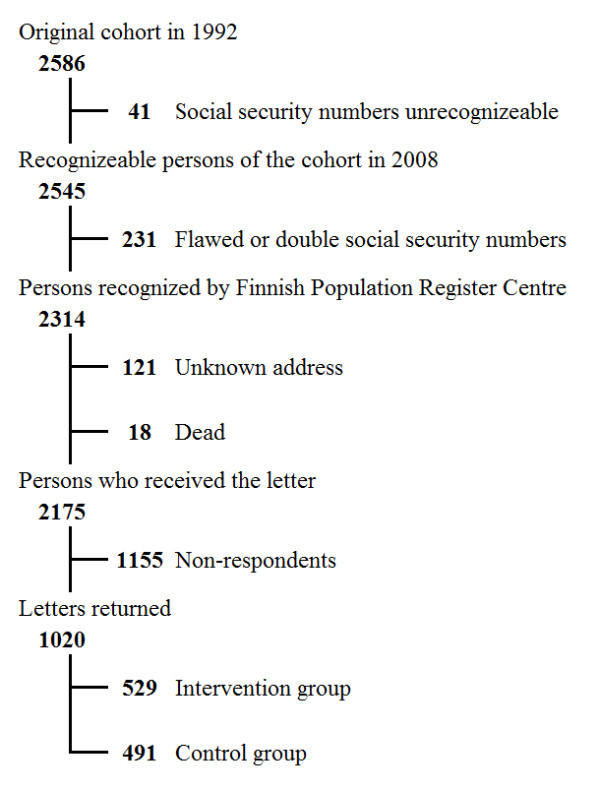
**Trial profile**.

The Ethical Committee of the Pirkanmaa Hospital District, Finland, approved the study protocol. The development of the cohort is presented in Figure 1. The cohort consisted of all subjects born in 1979 and living in 1992 in one of the previously mentioned four Ostrobothnian towns [14]. A questionnaire was sent to the available cohort (n = 2,175) to addresses obtained from the Population Register Centre. Intervention group indicates the part of the cohort born on an odd day and receiving one to four brief tobacco intervention(s) during annual school dental checkups. Control group indicates the part of the cohort born on an even day and having normal annual school dental checkups without brief tobacco interventions. The participation rates at the four initial sessions and this 2008 follow-up are presented in Table 1.

We used a postal questionnaire to assess smoking habits. In the envelopes there was also a cover letter describing the purpose and methodology of the study and a consent form. Recipients were requested to read, sign and return the consent form with the completed questionnaire. Only questionnaires returned with a signed consent form were used as data. The questions about smoking in the questionnaire were based on a questionnaire used and validated in twin studies [17]. The essential variable was whether or not the person was smoking at the time of the follow-up in 2008. Respondents were classified into three groups: smokers, ex-smokers and never-smokers. Those respondents answering "yes" to the question "Do you smoke?" (No/Yes) were classified as smokers. Those respondents answering "no" to the question "Do you smoke?", but answering "yes" to the question "During your life have you smoked over 5 packs of cigarettes or used an equivalent amount of tobacco in some other form?" were classified as ex-smokers. Those respondents answering "no" to both these questions were classified as never-smokers. Duration of smoking was calculated for ex-smokers by subtracting age at initiation from age at cessation. Duration of smoking was calculated for smokers by subtracting age at initiation from 29 (the average age of the cohort at the time of the questionnaire mailing).

We also measured some potential confounders for smoking. These were marital status, level of education and self-perceived health. Marital status was elicited (Single/Married/Cohabiting/Remarried/Divorced/Widowed). Single, divorced and widowed respondents were classified as single, while married, remarried and cohabiting respondents were classified as married or cohabiting in the analysis. Education was classified as higher education if the respondent had a polytechnic or university degree. All other education was classified as lower education. Respondents' self-perceived health was also elicited (Very good/Good/Average/Poor/Very poor/Can't say). After analysing the frequencies in each option the answers were reclassified as Very good/Not very good, where all answers other than Very good were classified as Not very good.

The analysis was performed using SPSS 16.0 and 17.0 for Windows. Frequencies and cross-tabulations were calculated. Associations between frequencies were tested using Pearson's χ2-test. Odds ratios (OR) in the logistic regression analysis for those in the intervention group being smokers compared to the control group were also calculated. A confidence interval of 95% was used in the odds ratios. Unpaired t-test was used to analyse respondent's age at initiation of smoking between the groups.

Written informed consent was obtained from the participants for publication of this manuscript. A copy of the written consent for is available for review by the Editor-in-Chief of this journal. This study was registered at http://clinicaltrials.gov (NCT01348646).

## Results

After one reminder the response rate was 46.9% (n = 1,020) (Table 1). There was no difference in response rate between the intervention group and the control group. Of the respondents in the intervention group 97.0% had received at least one brief intervention. Respectively 85.6%, 63.5% and 21.6% had received at least two, three or four brief interventions. There were no differences between the study groups by education, gender, marital status, self-perceived health or smoking rates during the initial study (Table 2). Those who were smokers in adolescence were as numerous in the responding as in the non-responding part of the cohort. There were no differences in baseline characteristics between the respondents and the non-respondents.

**Table 2:** Distribution of the measured confounders and smoking rates during the initial study in the intervention group and the control group*

	Control group		Intervention group	
	
	n	% of group	n	% of group
*Gender*				

Female	295	60.1	310	58.6

Male	196	39.9	219	41.4

*Marital status*				

Single	119	24.2	129	24.4

Married or cohabiting	372	75.8	400	75.6

*Education*				

Lower	171	35.1	177	33.7

Higher	316	64.9	348	66.3

*Self-perceived health*				

Very good	144	29.4	139	26.3

Other	345	70.6	390	73.7

*Smoking rate*				

In 1992	30	6.1	18	3.4

In 1993	44	8.9	45	8.6

*None of the differences between the study groups are statistically significant

Of all respondents 16.9% were smokers in 2008. Median age at initiation was 15 years. There was no difference between the groups in age at initiation. Of the intervention group and control group, 15.3% and 18.5% respectively were smokers. This difference of 3.2 percentage points in smoking was not statistically significant (*p *= 0.38). The odds ratio for being a smoker was not different between those in the brief intervention and the control group (OR 0.78, CI 0.56-1.09, *p *= 0.15). There was no difference in the share of ex-smokers or never-smokers between the intervention group and the control group. Furthermore, there was no difference in mean duration of smoking between the intervention group and the control group (median values 10 and 11 years respectively, *p *= 0.10). Repeating the brief intervention up to four times caused no change in smoking rates. There was no difference between groups in number of cigarettes smoked currently or before cessation.

The association between smoking and level of education was obvious; the prevalence of smoking was more than twice as high in the lower education group (*p *< 0.001). The brief intervention yielded no significant decrease in smoking in the group with higher education, but in the lower education group there was a significant difference of 12.3 percentage points between smoking prevalence in the intervention group and the control group (Table 3). A significant interaction effect was found between the brief intervention and education (*p *= 0.02). In the intervention group the odds ratio for being smokers was significantly less for those who had lower education (Table 4). When dividing the cohort by level of education and looking at the smoking rates of the group with lower education retrospectively, at the first follow-up in 1993, the share of smokers did not differ between the intervention group and the control group. In those with lower education later in life the share of smokers in 1993 was already greater than in those who later received higher education (16.4% vs. 4.8%).

**Table 3:** Distribution of never-smokers, ex-smokers and smokers in the intervention group and control group by gender, marital status, education and self-perceived health

		Never-smokers	Ex-smokers	Smokers	All		***p*-value**
		
		%	%	%	n	%	
*Gender*							

Female							0.48

	Intervention group	55.4	31.7	12.9	303	100	
	
	Control group	55.1	28.8	16.1	292	100	

Male							0.33

	Intervention group	45.5	35.7	18.8	213	100	
	
	Control group	38.3	39.4	22.3	188	100	

*Marital status*							

Single							0.72

	Intervention group	54.4	21.6	24.0	125	100	
	
	Control group	55.7	24.3	20.0	115	100	

Non-single							0.10

	Intervention group	50.4	37.1	12.5	391	100	
	
	Control group	46.3	35.6	18.1	365	100	

*Education*							

Lower							**0.04**

	Intervention group	39.7	38.5	21.8	174	100	
	
	Control group	32.9	32.9	34.1	164	100	

Higher							0.59

	Intervention group	57.1	30.8	12.1	338	100	
	
	Control group	56.7	33.3	9.9	312	100	

*Self-perceived health*							

Very good							0.60

	Intervention group	63.8	29.7	6.5	138	100	
	
	Control group	58.9	31.9	9.2	141	100	

Not very good							0.42

	Intervention group	46.8	34.7	18.5	378	100	
	
	Control group	44.1	33.4	22.5	338	100	

**Table 4:** Univariate odds ratios (OR) with 95% confidence intervals (CI) for smoking and ex-smoking in the subgroups by gender, marital status, education or self-perceived health

		OR (95% CI)	OR (95% CI)
		**for being smokers**	**for being ex-smokers**

*Female*			

	Control group	1.00	1.00
	
	Intervention group	0.77 (0.49-1.22)	1.14 (0.80-1.62)

*Male*			

	Control group	1.00	1.00
	
	Intervention group	0.86 (0.52-1.40)	0.84 (0.56-1.27)

*Marital status single*			

	Control group	1.00	1.00
	
	Intervention group	1.28 (0.69-2.36)	0.86 (0.47-1.57)

*Married or cohabiting*			

	Control group	1.00	1.00
	
	Intervention group	0.67 (0.45-1.01)	1.04 (0.78-1.41)

*Lower education*			

	Control group	1.00	1.00
	
	Intervention group	**0.55 (0.34-0.91)**	1.30 (0.83-2.04)

*Higher education*			

	Control group	1.00	1.00
	
	Intervention group	1.30 (0.79-2.14)	0.86 (0.62-1.20)

*Very good self-perceived health*			

	Control group	1.00	1.00
	
	Intervention group	0.75 (0.31-1.84)	0.95 (0.57-1.59)

*Not very good self-perceived health*			

	Control group	1.00	1.00
	
	Intervention group	0.80 (0.56-1.15)	1.01 (0.74-1.38)

The effect of the brief intervention was poor in both males and females (Tables 3 and 4). There was no difference in the effectiveness of the brief intervention when those with very good self-perceived health were compared to others.

## Discussion

We found no true effect in adulthood smoking gained through brief tobacco intervention(s) performed in adolescence. The difference between the test and control groups is similar to that observed in the earlier study (3.2% more smokers in the control group) but remains small and questionable. Single or repeated brief tobacco intervention(s) have been reported to have an effect on smoking in short (6-12 months) follow-ups [18-21]. Our results suggest that this effect may not be long-lasting. However, populations and intervention settings differ from the one we studied.

In our method we tried to increase the impact of the brief intervention by the use of photographs and the subjective experience of seeing one's own mouth in a mirror. It could be speculated that the intervention effect should be greater with the method we used. We found no references in earlier tobacco intervention studies stressing the cosmetic impact of smoking. Possibly the brief interventions could have been more effective if they had been repeated with some new content every time.

The brief intervention did have an effect on the lower education group, but the practical significance of this effect is unclear. Higher education is a predictor of smoking cessation [22] and there were clearly fewer smokers in the group with higher education. However, the brief intervention had no effect among respondents with higher education. This suggests that the anti-smoking effect of the brief intervention was weaker than the protective effect of higher education. The importance of adequate education for smoking prevention has been shown in districts where education is poor [23]. We know that those who do not complete higher education later in life are more likely to smoke already in adolescence. How could we find these adolescents who are prone to smoking in adulthood and make an effective intervention at the right time to prevent this? It is no longer a norm in Finland that children from less educated parents are also less educated.

This study has several limitations. One of the most important limitations is a decidedly high drop-out rate. This is an unavoidable consequence of the long follow-up time, which we tried to avoid by sending a reminder letter to those who did not answer the first letter. It is difficult to keep participants interested in continuing until the end of the trial and such long follow-up studies are uncommon. The use of incentives could have produced an increase in response rate [24]. Although there was a similar portion of respondents in the intervention group and the control group, small differences are easily statistically non-significant in small populations and the possibility of beta error is present. There was no difference between the intervention group and the control group except the brief intervention(s), thus any difference in their smoking may be seen as a result of the brief tobacco intervention. It is not obvious that a subsequently found difference or its absence can be attributed to the intervention. This is a limitation of our interpretations.

The cohort was not randomized but divided into two groups by date of birth (odd/even day of the month). This method does not have the validity of random numbers, but it is unlikely to have caused any bias in the study. We did not determine possible periods of abstinence and their duration for the ex-smokers or smokers, so their actual smoking time may be less than our calculations suggest. It is possible that smokers were less keen to respond to postal questionnaires about smoking. This is supported by the high drop-out rate of frequent smokers after the first round of the initial study [14]. There was evidence that the brief intervention was sometimes performed inadequately, thus with additional training of personnel the impact could have been more marked [16].

The participants in the control group were not specifically informed about the measures provided to the intervention group during dental checkups, but there were children from both groups in the same schools and classes. This is a limitation of our study when considering the informational part of the brief intervention. However, the subjective impression with the photographs of dental discoloring and one's own mouth seen through a mirror could not be transferred to another participant. It is also possible that those who did not respond to the postal questionnaire were living in different areas than those who did respond. Furthermore, it is possible that the group that did not respond had a different education spectrum from those who responded. This may have produced bias in the study. We used a self-perceived health measure to assess the subjective quality of life of the respondent. This measure is a good predictor of mortality [25], but is non-objective and narrow. However, subjectively perceived health is an important motivator in a person's health behavior; those who perceive their health to be excellent do not find it necessary to make changes in their behavior.

A strength of this study is that it brings new information about the long-term effectiveness of brief intervention in smoking prevention. Unfortunately, we cannot tell what made this brief intervention unsuccessful. The brief intervention of this study focused mainly on cosmetic effects in oral health and was fuelled by the adolescents' wish to retain a good appearance. Focusing on the most obvious severe effects of smoking (cancers, COPD etc.) is possibly not effective when attempting to prevent adolescent smoking, because adolescents rarely see themselves as still smoking in adulthood. It has been shown that school dentists are motivated to prevent adolescent smoking, but only one in five regularly intervenes in young patients' smoking [26]. All health care professionals should understand their important role in smoking prevention.

There is evidence that adolescents with insufficient skills in managing negative and hostile emotions could be more prone to start smoking [27]. Teaching these skills to adolescents might have a diminishing effect on their smoking. If a person does not start smoking in adolescence, he is very unlikely to start smoking later in life [28]. This makes the effort to prevent adolescent smoking worthwhile. The costs to society of smoking are heavy and it is essential to find suitable methods to prevent the initiation of smoking. Since the resources for preventive health care are limited, they must be directed to actions that have a proven effect.

## Conclusions

The results of this study suggest that the long term effect of brief tobacco intervention supported by cosmetic approach and performed in adolescence by a dentist is likely to be small. Repeating the brief intervention up to four times seems to bring no additional benefit in the long-term follow-up.

## Competing interests

The authors declare that they have no competing interests.

## Authors' contributions

AJS gathered and processed the data and wrote the paper. JK designed the study and wrote the paper. KJM supervised, designed the study, processed the data and wrote the paper. All authors read and approved the final manuscript.
